# Association of working hours and cumulative fatigue among Chinese primary health care professionals

**DOI:** 10.3389/fpubh.2023.1193942

**Published:** 2023-05-25

**Authors:** Yushi Lu, Zhi Li, Qingsong Chen, Yuting Fan, Jin Wang, Yonghao Ye, Yongqi Chen, Tian Zhong, Ling Wang, Ying Xiao, Dongmei Zhang, Xi Yu

**Affiliations:** ^1^Faculty of Medicine, Macau University of Science and Technology, Macau, Macao SAR, China; ^2^School of Public Health, Guangdong Pharmaceutical University, Guangzhou, Guangdong, China; ^3^Chinese Center for Disease Control and Prevention, National Institute of Occupational Health and Poison Control, Beijing, China; ^4^Resproly Pharmaceutical Technology Co. Ltd, Zhuhai, Guangdong, China; ^5^Guangdong-Hong Kong-Macau Joint Laboratory for Contaminants Exposure and Health, Guangzhou, Guangdong, China

**Keywords:** working hours, cumulative fatigue, occupational stress, primary health care, mediating effect

## Abstract

**Introduction:**

The association between long working hours and cumulative fatigue is widely acknowledged in the literature. However, there are few studies on the mediating effect of working hours on cumulative fatigue using occupational stress as a mediating variable. The present study aimed at investigating the mediating role of occupational stress in the relationship between working hours and cumulative fatigue in a sample of 1,327 primary health care professionals.

**Methods:**

The Core Occupational Stress Scale and the Workers’ Fatigue Accumulation Self-Diagnosis Scale were utilized in this study. The mediating effect of occupational stress was examined using hierarchical regression analysis and the Bootstrap test.

**Results:**

Working hours were positively associated with cumulative fatigue via occupational stress (*p* < 0.01). Occupational stress was found to partially mediate the relationship between working hours and cumulative fatigue, with a mediating effect of 0.078 (95% CI: 0.043–0.115, *p* < 0.01), and the percentage of occupational stress mediating effect was 28.3%.

**Discussion:**

Working hours can be associated with cumulative fatigue either directly or indirectly via occupational stress. As a result, by reducing occupational stress, primary health care professionals may reduce the cumulative fatigue symptoms caused by long hours of work.

## 1. Introduction

It is a very serious problem about the shortage of personnel in China’s primary health care system, which has added a significant workload to the primary health care professionals (1). Furthermore, the main duty of these employees is to provide healthcare services to patients and the public. Primary health care workers often have longer working hours than other occupational groups to ensure prompt service delivery and nursing work. They are also more likely to work overtime. Survey results indicate that over half (54%) of registered nurses in the United States work over 39 h per week, and 19.3% work 48 h or more per week (2). Long working hours have not yet been defined uniformly across nations. In the EU, workers’ weekly working hours, including overtime hours, that exceed 48 h are considered long working hours (3). In South Korea, the Labor Standards Act defines long working hours as exceeding 52 h per week (4). And in China, the Labor Law provides in Article 36 that the country implements a working hour system in which laborers work no more than 8 h per day and no more than 44 h per week on average, and working more than 8 h per day or more than 44 h per week is defined as long working hours (5). Another study examining the working hours of doctors in Guangdong Province, China found that 68.5% of them worked more than 40 h per week (6). A sizable number of studies have shown that extended working hours can have negative impacts on the health of professional workers (7). A sizable number of studies have shown that extended working hours can have negative impacts on the health of professional workers (8). The generation of fatigue, including cumulative fatigue, is the most common, and the longer the working hours, the greater the degree of cumulative fatigue (9). Cumulative fatigue in professional groups is a condition in which an individual suffers from physical overwork, long-term emotional stress, or a lack of sleep, resulting in a decline, deteriorating health, emotional disturbance, or a decrease in work efficiency (10). Long hours of work have been found to have a greater impact on the professional population’s cumulative fatigue, and limiting excessively long working hours may help alleviate the negative effects of accumulated fatigue (11).

There was a period of normalization of epidemic prevention and control in China during the investigation and study period of September 2021 to December 2022, and primary healthcare professionals played an important role in responding to the new Coronavirus (12). Professionals of primary health care systems were responsible for basic work, as well as epidemic prevention and control during this time. Basic work includes community health education, monitoring chronic disease and infectious disease, taking care of older adult patients, managing vaccination, and more. Epidemic prevention and control work includes virus screening, public place disinfection, and health education to prevent COVID-19. These strict normalization policy for epidemic prevention places a significant burden on primary health care professionals, such as an increasing number of outpatient visits, resulting in extended working hours (13). Overtime working occurs frequently, causing professionals to experience increased fatigue and occupational stress (14). According to some studies, working hours are linked to symptoms like occupational stress and cumulative fatigue, and occupational stress have an intermediary effect on working hours and cumulative fatigue (15). Occupational stress, also known as occupational pressure, is an adverse reaction caused by workplace requirements and career duties that exceed the ability of the occupational group, including physiological and psychological reactions (16). The researchers found that long-term occupational stress could negatively impact physical health, such as hormone imbalance and hypertension. And it also causes mental health damage, such as depression (17). According to some studies, relieving the professionals’ occupational stress will reduce fatigue caused by long-term work (18). Therefore, this study discussed how primary health care professionals’ occupational stress affected the role of working hours in cumulative fatigue and proposed measures to address the cumulative fatigue and occupational stress of the primary health care professionals.

Previous studies have examined the relationship between the professional population’s cumulative fatigue and working time, as well as the effects of occupational stress and cumulative fatigue on employees. It has also been found that working time and occupational stress are significantly correlated (19). However, no studies have examined the impact of occupational stress as a mediating variable between working time and cumulative fatigue. Furthermore, previous research did not consider the primary health care professionals as research target. This study addressed the above limitations, including the lack of considerations on mediation factors and sampling specificity, by thoroughly investigating the relationship between working time, cumulative fatigue, and occupational stress among primary health care system professionals. The study also investigated the role and mechanism of occupational stress as mediating variables at primary health care professionals between working time and cumulative fatigue. It is anticipated that this study will examine primary health care professionals’ working time, cumulative fatigue, and occupational stress; second, to find out how working hours are related to cumulative fatigue, occupational stress among primary health care professionals. And then, to investigate how, as a mediating variable, the occupational stress of primary health care professionals influences the effect of working hours on cumulative fatigue. After that, to present ideas on how to solve the cumulative fatigue of professionals in the primary health care system, particularly how to alleviate the cumulative fatigue by addressing the symptoms of occupational stress of professionals when working hours cannot be reduced.

## 2. Methods and materials

### 2.1. Participants

This was a cross-sectional study that began in September 2021 and lasted until December 2021. The survey was conducted in Guangdong Province, China, using a multi-stage stratified sampling method. According to the Guangdong Provincial Bureau of Statistics, the Gross Domestic Product (GDP) of each prefecture-level city was divided into three parts: economically good (more than 125 billion), economically medium (28–125 billion), and economically poor (less than 28 billion). Each GDP level was assigned four primary medical and health institutions at random, including district-level health bureaus, district-level centers for disease control and prevention, community health service centers, and public hospitals below the district level. All doctors, nurses, and medical technicians at the selected primary health institutions were surveyed. Over a week, the WeChat app was used to survey every employee of the institution online. Following the completion of the questionnaire by survey respondents, a staff member in each primary medical and health institution would be responsible for collecting the questionnaire information and reporting it to data processing personnel. This research object’s inclusion criteria were: the age was over 18 years old, and participant had been continuously working on current position for more than half a year. The survey included all employees from 12 primary health institutions in 10 cities, for a total of 1,430 people (*n* = 1,430). Upon completion of the survey, a total of 1,327 questionnaires were deemed eligible for analysis, resulting in a commendable response rate of 92.8%.

### 2.2. Basic investigation

Participants’ investigation information was collected via online questionnaires, including basic information and occupation information. The basic information was age, gender, marital status, and education level. And the occupation information was personal monthly income, whether they are on duty, and whether they work night shift. Respondents’ daily working hours were inquired about in terms of working hours. There is currently no agreed-upon definition of long working hours. This study refers to laws and policies such as China’s “People’s Republic of China Labor Law,” “Regulations on Working Hours of State Council Employees,” and related concepts of overtime work in the International Labor Organization (ILO). Working more than 8 h per day or 44 h per week is considered excessive (5). The related variable invested in this study is the working hours per day of the research participants.

### 2.3. Measurement of cumulative fatigue

The “Self-diagnosis Questionnaire for Workers’ Fatigue Accumulation Degree” developed by the Japanese Ministry of Health, Labor, and Welfare was used to assess cumulative fatigue in this study (20). This scale is used to assess the accumulation of fatigue and overwork in the occupational population. Researchers in China have used this scale to measure the fatigue of the subjects in previous studies, and it is widely used. Previously, some Chinese researchers applied this scale to a machinery manufacturing plant, investigated the overwork status of factory workers, and proposed preventive measures for employee overwork (21).

The “evaluation of subjective symptoms” and the “evaluation of work conditions” dimensions comprise the Self-Diagnostic Questionnaire of Workers’ Fatigue Accumulation Level. These two dimensions each have 13 entries and 7 entries, for a total of 20 entries. The scores of the 13 items were added up in the “Assessment of subjective symptoms” dimension, and the total score was divided into four grades, with a total score of less than 5 being grade I and a total score of 5 to 10 being grade II. A total score of 11 to 20 is considered grade III, while a total score of more than 20 is considered grade IV. In the dimension “Working Status Evaluation,” the scores of the seven items are added up and divided into four grades. A total score of 0 represents grade A, a total score of 1–2 represents grade B, a total score of 3–5 is graded as C, and a score of more than 5 is graded as D. Subsequently, the cumulative fatigue score is calculated using the “Work Burden Score Scale” in conjunction with the classification of the two dimensions. The level of cumulative fatigue score shows the degree of fatigue in the occupational group. When the score reaches 2 points, it means that the employee has symptoms of cumulative fatigue. The reliability test results of this scale: the Cronbach’s *α* coefficients for the total scale and the two dimensions of this scale are 0.892, 0.895, and 0.711, respectively. The validity test includes 20 items on the scale, and the results are as follows: The KMO was 0.921, the Bartlett sphericity test 2 value was 9,981.76 (*p* < 0.01), the cumulative variance contribution rate of the scale’s common factor was 51.07%, and the factor loading value of each item ranged from 0.439 to 0.852.

### 2.4. Measurement of occupational stress

The “Core Occupational Stress Scale” (COSS) developed by the Chinese Center for Disease Control and Prevention and the Institute of Poison Control was used to assess occupational stress levels in this study (22). This scale has been used in a survey of occupational groups in China as a tool to measure employee occupational stress, and the results have been positive. There are four dimensions of COSS: “social support,” “organization and reward,” “demand and effort,” and “autonomy.” It has 17 items when added up from the four dimensions. The COSS employs the Likert 5-point scoring methodology, whereby respondents are presented with five response options ranging from “completely disagree” (1 point) to “disagree” (2 points), “basically agree” (3 points), “agree” (4 points), and “strongly agree” (5 points). The dimensions “social support” and “autonomy” use the reverse scoring method, while the dimensions “organization and return” and “requirement and effort” use the forward scoring method. Finally, the total occupational stress score is calculated by adding the scores of the 17 items in the four dimensions. The level of occupational stress score indicates the employee’s level of occupational stress, and a score of occupational stress above 50 indicates that the employee has occupational stress. The reliability test results of the “Core Occupational Stress Scale” showed that the Cronbach’s *α* coefficients of the total scale and the four dimensions were 0.681, 0.882, 0.754, 0.841, and 0.832, respectively. The scale comprised 17 items, and the statistical results yielded a KMO measure of 0.835, indicating an adequate sample size for factor analysis. Additionally, the Bartlett’s test of sphericity achieved a significant value of 9,541.23 (*p* < 0.01), indicating that the correlation structure between the items was suitable for factor analysis. Furthermore, the factor loading coefficients of each item ranged from 0.488 to 0.922, indicating a satisfactory level of item convergence.

### 2.5. Statistical analysis

**Figure 1:**
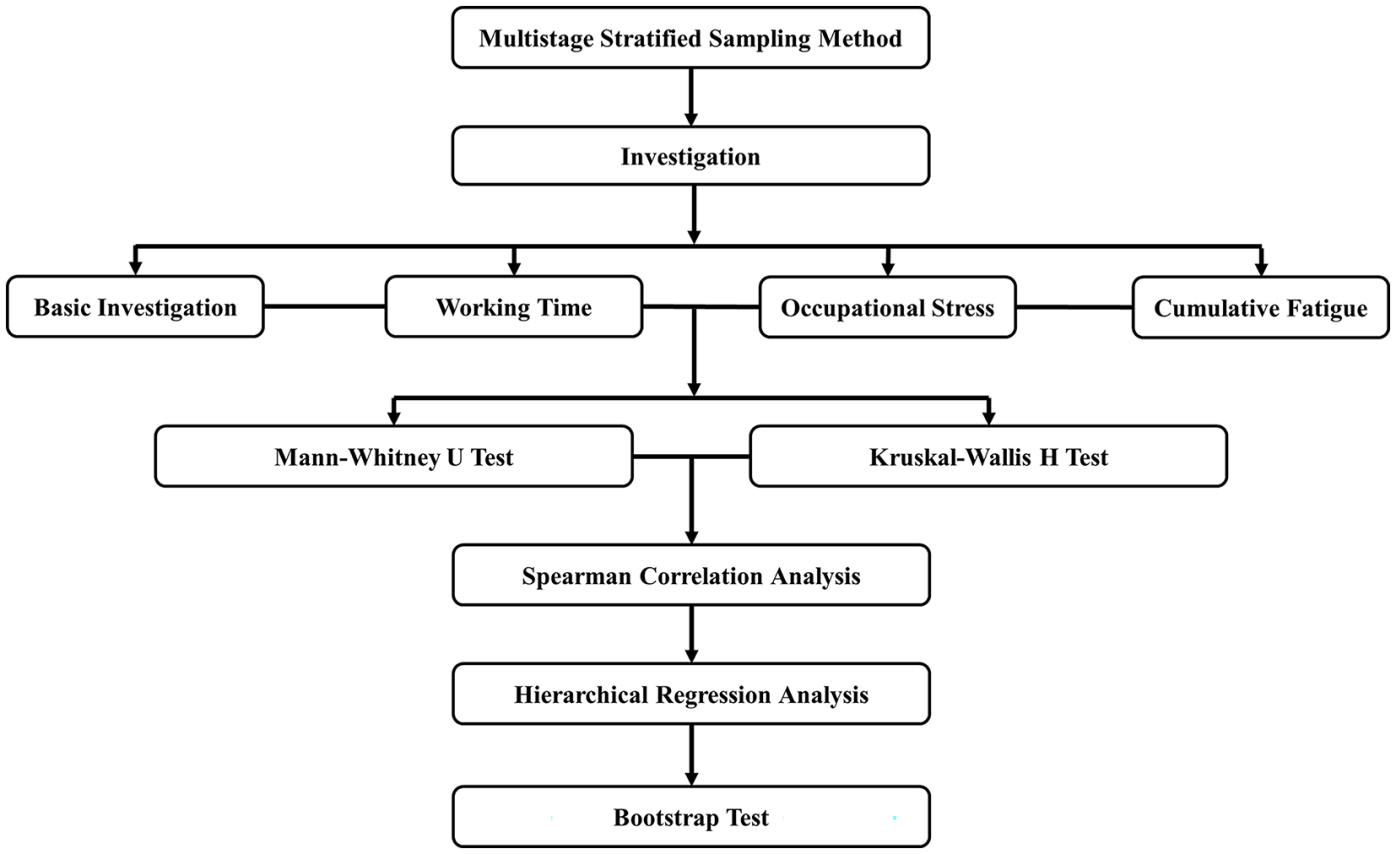
The methodology framework of investigation and statistics.

Following the collection of questionnaire information from each institution, data processing personnel screened the questionnaires and classified those with a missing item rate greater than 20% as invalid questionnaires, while including the remaining questionnaires as valid questionnaires in the database. Epi Data version 3.1 software was used for data entry. To avoid errors during quality control, two people entered data in parallel. SPSS version 22.0 software (IBM, Armonk, NY, United States) was used for data analysis. Since the normal distribution was not satisfied when the data was tested for normality, the median (Q1, Q3) was used for descriptive statistics. The Mann–Whitney U test was used to determine the significance of two graded variables: “gender,” “education level,” “marital status,” “whether shift work,” and “whether work night shift.” The Kruskal–Wallis *H* test was used to test the significance of multiple graded variables, including “age,” “*per capita* monthly income,” and “position.” The correlation between working hours, occupational stress, and cumulative fatigue was then examined using Spearman correlation analysis. In the next step, hierarchical regression analysis is used, with cumulative fatigue as the dependent variable, and basic conditions, daily working hours, and occupational stress as independent variables into the mediation effect model to analyze the effect of each link on the dependent variable. Finally, to test the mediating effect of occupational stress between working hours and cumulative fatigue, Model 4 was used in the Process 4.1 plug-in in SPSS for the Bootstrap method (23). The predictor variable is the daily working hours of employees in the primary health system, the outcome variable is the employees’ cumulative fatigue score, the mediator variable is the employees’ occupational stress level, and the control variables are age, education level, occupation, shift work, and whether to work night shift. The total effect of working hours on cumulative fatigue is divided into direct and indirect effects in the model. The total effect refers to the effect of the predictive variable employee’s working hours on the outcome variable cumulative fatigue when the mediator variable occupational stress is not controlled. The direct effect is the effect of the predictive variable employee’s working hours on the outcome variable cumulative fatigue when the mediator variable cumulative fatigue is controlled. The indirect effect refers to the effect of the predictive variable employee’s working hours on the outcome variable cumulative fatigue through the mediating variable occupational stress, also known as the mediating effect. Two-tailed test level *α* = 0.05. The methodology framework of investigation and statistics is shown in Figure 1.

## 3. Results

### 3.1. Basic information of participants

Among all research subjects, 28.5% were men and 71.5% were women; 31.3% were under the age of 30, 36.5% were between the ages of 31 and 39, and the remaining 32.2% were over the age of 40. In the occupational survey, 32.3% of the participants were doctors, 41.3% were nurses, and 26.4% were medical technicians. Of all participants, 43.9% of health system workers worked shifts, while 41.4% worked night shifts (Table 1).

**Table 1:** Characteristics and scores of working time, cumulative fatigue, and occupational stress among participants.

Variables	Counts (Ratio/%)	Working hours				Occupational stress	Cumulative fatigue		
		Score	Z/H	*p*	Total/Score	Z/H	*p*	Total/Score	Z/H	*p*
Gender			−2.144	0.032		−0.365	0.715		−1.633	0.102
Male	378 (28.5)	8.0 (8.0,10.0)			44.5 (39.0,50.0)			2.0 (0.0,4.0)		
Female	949 (71.5)	8.0 (8.0,9.0)			45.0 (40.0,50.0)			2.0 (0.0,4.0)		
Age/Year			2.041	0.360		4.301	0.116		19.806	**<0.001**
≤30	416 (31.3)	8.0 (8.0,9.0)			45.0 (39.0,50.0)			2.0 (0.0,4.0)		
31–40	484 (36.5)	8.0 (8.0,9.0)			45.0 (40.0,51.0)			2.0 (0.0,4.0)		
≥41	427 (33.2)	8.0 (8.0,9.0)			44.0 (39.0,49.0)			1.0 (0.0,4.0)		
Education level			−2.607	**0.009**		−0.760	0.448		−3.721	**<0.001**
Junior college or below	634 (47.1)	8.0 (8.0,9.0)			45.0 (40.0,50.0)			2.0 (0.0,4.0)		
Bachelor or above	693 (52.9)	8.0 (8.0,9.0)			44.0 (39.0,50.0)			2.0 (0.0,4.0)		
Marital status			−0.623	0.533		−0.492	0.623	c	−1.442	0.149
Unmarried	329 (24.8)	8.0 (8.0,9.0)			45.0 (39.0,50.0)			2.0 (0.0,4.0)		
Married	998 (75.2)	8.0 (8.0,9.0)			45.0 (40.0,50.0)			2.0 (0.0,4.0)		
Monthly income/USD			3.198	0.202		17.406	**<0.001**		1.053	0.591
≤700	488 (36.8)	8.0 (8.0,9.0)			46.0 (41.0,51.0)			2.0 (0.0,4.0)		
701–999	412 (31.0)	8.0 (8.0,9.0)			45.0 (40.0,50.0)			2.0 (0.0,4.0)		
≥1,000	427 (32.2)	8.0 (8.0,9.0)			43.0 (38.0,49.0)			2.0 (0.0,4.0)		
Occupation			14.512	**0.001**		10.574	**0.005**		20.079	**<0.001**
Doctor	428 (32.3)	8.0 (8.0,10.0)			46.0 (40.0,51.8)			2.0 (0.3,4.0)		
Nurse	548 (41.3)	8.0 (8.0,9.0)			45.0 (40.0,50.0)			2.0 (0.0,4.0)		
Medical technician	351 (26.4)	8.0 (7.0,9.0)			44.0 (39.0,49.0)			1.0 (0.0,4.0)		
Shift or not			−3.857	**<0.001**		−4.644	**<0.001**		−4.642	**<0.001**
No	745 (56.1)	8.0 (7.0,9.0)			44.0 (39.0,49.0)			2.0 (0.0,4.0)		
Yes	582 (43.9)	8.0 (8.0,9.0)			46.0 (40.0,51.0)			2.5 (0.0,4.0)		
Night shift			−6.816	**<0.001**		−4.606	**<0.001**		−9.112	**<0.001**
No	777 (58.6)	8.0 (7.0,9.0)						1.0 (0.0,4.0)		
Yes	550 (41.4)	8.0 (8.0,10.0)						3.0 (1.0,4.3)		

The bold values means *p* < 0.05.

### 3.2. Factors of working hours, cumulative fatigue, and occupational stress

Employees in the primary health care system worked an average of 8.5 h per day, with 33.5% of working hours being excessive. According to the analysis results, there was a statistically significant difference in the working hours of primary health care professionals from various educational backgrounds and occupations (*p* < 0.05).

The average occupational stress score among employees in the primary health care system was 45.0 (40.0, 50.0), with a 27.5% detection rate. Furthermore, the occupational stress level of primary health care workers who must work shifts and night shifts was significantly higher than that of workers who worked during normal business hours (*p* < 0.05).

The average cumulative fatigue score of primary health system staff was 2.0 (0.0, 4.0), with cumulative fatigue accounting for 57.5% of all staff. The results showed that there seemed to be statistically significant differences in the cumulative fatigue degree of primary health care workers of various ages, education levels, and occupations (*p* < 0.05). The cumulative fatigue was more severe among primary health care professionals under the age of 30, with a bachelor’s degree or higher, and who are doctors. At the same time, the cumulative fatigue of primary health care workers who must work shifts and nights was significantly higher than that of workers who worked regular hours (*p* < 0.05). Details are shown in Table 1.

### 3.3. Correlation of working hours, occupational stress, and cumulative fatigue

The results of Spearman correlation analysis showed that primary health care professionals’ working hours are positively correlated with occupational stress and cumulative fatigue. Occupational stress and cumulative fatigue symptoms were more severe in primary health care workers who work longer hours (*r* = 0.190, 0.365, *p* < 0.01). At the same time, occupational stress among primary health care professionals was found to be positively related to cumulative fatigue, with the higher the occupational stress score, the more severe the symptoms of cumulative fatigue (*r* = 0.546, *p* < 0.01). Details are given in Table 2.

**Table 2:** Correlation analysis of working time, occupational stress, and cumulative fatigue.

Variables	Working hours	COSS	Cumulative fatigue
Working hours	1.000		
COSS	0.190^**^	1.000	
Cumulative fatigue	0.365^**^	0.546^**^	1.000

PS: ^**^means *p* < 0.01, and COSS means the Core Occupational Stress Scale, the same as below.

### 3.4. Stratified regression analysis on working hours, occupational stress, and cumulative fatigue

According to previous studies, education level, occupation, and shift work may be the confounders, which need to be considered in the research (24–26). And with the findings in Table 1, among the basic information of employees in the primary health care system, the factors that significantly affect cumulative fatigue include the employees’ age, education level, occupation, shift, and night shift. The tolerance range of each factor was 0.583–0.969, and the variance inflation factor (VIF) ranges from 1.032–1.714, according to the multicollinearity analysis of these factors. As a result, there was no collinearity between the variables, and hierarchical regression analysis can be performed. Using cumulative fatigue score as the dependent variable, the first layer of the regression model included confounders as control variables, such as “age,” “education level,” “occupation,” “whether shift work” and “whether work night shift.” Based on the first step, “working hours” was included as a second-level variable in the regression model in the second step. Working hours were found to be positively associated with cumulative fatigue, and the variance explanation for cumulative fatigue symptoms was 7.7%. The scores of the four dimensions of occupational stress were then included in the regression model as the third layer of variables based on the second step. The findings revealed that the scores of “demand and effort” and “organization and reward” were positively correlated with the cumulative fatigue of employees, while the scores of “social supports” and “autonomy” were negatively associated with the cumulative fatigue scores of employees in the primary health system, with occupational stress accounting for 26.2% of the variation in accumulated fatigue, as shown in Table 3.

**Table 3:** Stratified regression analysis on working hours, occupational stress, and cumulative fatigue.

Variables	Block 1		Block 2		Block 3	
	*β*	VIF	*β*	VIF	*β*	VIF
Age/Year (≤30 as reference)						
31–40	0.037	1.561	0.037	1.561	−0.014	1.585
≥41	−0.042	1.539	−0.042	1.539	−0.071**	1.567
Education level (Junior college or below as reference)						
Bachelor or above	0.082**	1.182	0.068*	1.184	0.058*	1.227
Occupation (doctor as reference)						
Nurse	−0.053	1.474	−0.039	1.476	−0.013	1.491
Medical technician	−0.094**	1.400	−0.073*	1.406	−0.020	1.424
Shift or not (shift as reference)						
No shift	−0.066	1.771	−0.059	1.771	−0.052	1.846
Night shift or not (night shift as reference)						
No night shift	0.284**	1.722	0.235**	1.752	0.199**	1.758
Working hours			0.282**	1.034	0.149**	1.135
Occupational stress						
Social support					−0.214**	1.174
Organization and reward					0.071*	1.385
Demand and effort					0.406**	1.380
Autonomy					−0.071**	1.079
*F*	18.459		121.315		150.340	
Adjusted *R^2^*	0.084		0.161		0.423	
*ΔR^2^*	0.089		0.077		0.262	

PS: *means *p* < 0.05, **means *p* < 0.01 (two-tailed).

### 3.5. Mediation of occupational stress between working hours and cumulative fatigue

The results in Table 3 showed that the predictive variable working hours had a significant predictive effect on the outcome variable cumulative fatigue (*t* = 7.468, *p* < 0.01), and when the mediator variable occupational stress was put in, the predictive variable working hours still had a significant predictive effect on the outcome variable cumulative fatigue (*t* = 6.170, *p* < 0.01). In this model, whether the predictor variable was included in the 95% confidence interval of Bootstrap was used to determine whether there was a mediating effect. As shown in Tables 4, 5, the upper and lower limits of the 95% confidence intervals for the direct effect of primary health system employees’ working hours on cumulative fatigue and the mediating effect of occupational stress do not include 0, indicating that professionals’ working hours could not only predict cumulative fatigue directly, but also indirectly through the mediating role of occupational stress. The direct and mediating effects were 0.198 (95% CI: 0.135–0.261, *p* < 0.01) and 0.078 (95% CI: 0.043–0.115, *p* < 0.01), respectively, and the percentage of occupational stress was 28.3%.

**Table 4:** The test results of occupational stress in the mediation model between working hours and cumulative fatigue.

Outcome variable	Predictor variable	Fit index		Coefficient significance	
		*R* ^2^	*F*	*t*	*β*
Cumulative fatigue	Working hours	0.193	45.102	7.468	0.276**
Occupational stress	Working hours	0.072	14.509	4.169	0.633**
Cumulative fatigue	Occupational stress	0.400	109.657	21.290	0.123**
	Working hours			6.170	0.198**

PS: **p < 0.01 (two-tailed).

**Table 5:** Decomposition table of total effect, direct effect, and mediation effect.

Variables	Effect value	Standard error of indirect effect	95% confidence interval		Relative effect value
			Lower limit	Upper limit	
Total effect	0.276	0.006	0.131	0.154	
Indirect effect	0.078	0.018	0.043	0.115	28.3%
Direct effect	0.198	0.032	0.135	0.261	71.7%

To determine if there was a difference in the mediating effect across groups with various degrees of occupational stress, participants were divided into two groups based on the median: low occupational stress (COSS score less than 45) and high occupational stress (COSS score higher than or equal to 45). The influence of work stress as a moderator was evaluated between the two groups. As shown in Table 6. In the high occupational stress group, the direct effect and mediating effect values were 0.218 (95% CI: 0.140–0.295, *p* < 0.01), 0.037 (95% CI: 0.008–0.071, *p* < 0.01), and the mediating effect percentage was 14.37%. In the low occupational stress group, the direct and mediating effect values were 0.342 (95% CI: 0.249–0.434, *p* < 0.01), 0.030 (95% CI: 0.010–0.052, *p* < 0.01), and the mediating effect percentage was 8.06%. The mediating effect of occupational stress was greater in the high occupational stress group, and the effect of alleviating cumulative fatigue caused by working hours was better by reducing occupational stress.

**Table 6:** Decomposition table of total effect, direct effect, and mediation effect among professionals with different occupational stress levels.

COSS	Variables	Effect value	Standard error of indirect effect	95% confidence interval		Relative effect value
				Lower limit	Upper limit	
High level	Total effect	0.254	0.042	0.171	0.337	
	Indirect effect	0.037	0.164	0.008	0.071	14.37%
	Direct effect	0.218	0.040	0.140	0.295	85.63%
Low level	Total effect	0.372	0.048	0.274	0.466	
	Indirect effect	0.030	0.011	0.010	0.052	8.06%
	Direct effect	0.342	0.047	0.249	0.434	91.94%

**Figure 2:**
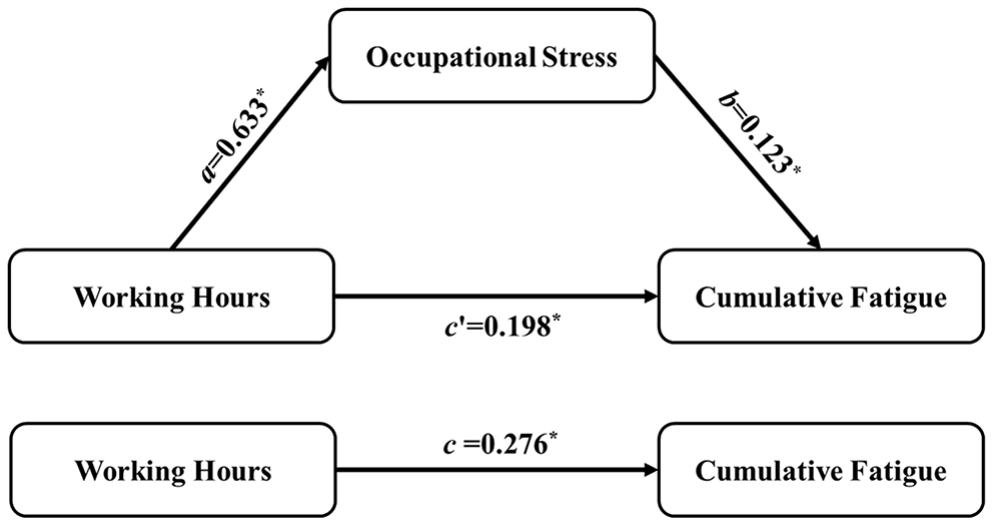
The mediating model of occupational stress between working hour and cumulative fatigue.

Figure 2 depicts the mediation effect’s path map. Among them, c represented the total effect of the predictor variable working hours on the outcome variable cumulative fatigue, with a value of 0.276 (*p* < 0.05); a represented the predictor variable working hours on the mediator variable occupational stress, with a value of 0.633 (*p* < 0.05); b represented the effect of the mediator variable occupational stress on the outcome variable cumulative fatigue, with an effect value of 0.123 (*p* < 0.05); c’ was the direct effect of the predictor variable working hours on the outcome variable cumulative fatigue after introducing the mediator variable occupational stress, with an effect value of 0.198 (*p* < 0.05).

## 4. Discussion

To sum up, the primary health care professionals’ average daily working time was 8.5 h, with a work overtime rate of 33.5%. An average occupational stress score was 45.0 (40.0, 50.0), with 27.5% participants having occupational stress symptoms. And the average score of professionals’ cumulative fatigue was 2.0 (0.0, 4.0), with a 57.5% detection rate. The primary health care professionals’ working hours were positively associated with cumulative fatigue. And the professionals’ working hours were also positively associated with cumulative fatigue through the mediation effect of occupational stress.

### 4.1. Effect of working hours on cumulative fatigue

According to the findings of this study, 33.5% of professionals in the primary health care system work more than 8 h per day. Doctors or those with higher education work longer hours daily. Furthermore, primary health care employees who require shifts and night shifts have significantly longer daily working hours than those who do not (*p* < 0.05). Due to the specific characteristics of their jobs, health care professionals need to provide care to patients around the clock, so atypical working hours have become the norm for medical staff (27). Especially for primary doctors, some researchers surveyed that the average working hours of doctors across the United States was 52.2 h per week, which exceeded the standard for the longest working hours (28). Furthermore, Japanese researchers conducted a survey of cardiovascular doctors’ working hours, and the results revealed that 75.5% of doctors worked more than 60 h per week (29). At the same time, the study’s findings indicate that primary health care professionals with a bachelor’s degree or higher, who are doctors and must work shifts and night shifts, have longer working hours, which is consistent with the findings of other researchers.

According to the findings of this study, 57.5% of professionals in the primary health care system have cumulative fatigue, which means that more than half of them have symptoms of cumulative fatigue. The study’s findings revealed that the likelihood of cumulative fatigue symptoms was higher among primary health system workers who were younger, had higher education, were doctors, and required shift and night shifts, which was consistent with the findings of other researchers (30, 31). Furthermore, the proportion of cumulative fatigue measured in this study is higher than the proportion of work fatigue measured by Zhan Y. X. et al. using the Fatigue Scale-14 scale (35.06%) (32), as well as that of Dyrbye L. N. et al. using the Maslach Burnout Inventory (MBI) Human Results Services Survey (35.3%) (33).

According to the findings of the preceding study, professionals in the primary health care system work longer hours and experience more severe cumulative fatigue symptoms. Due to the broad scope of work in the primary health system, many tasks are heavy, and the probability of cumulative fatigue among professionals also increases. Overtime work has become an unavoidable phenomenon in the healthcare industry. Furthermore, because of the recent COVID-19 pandemic, many employees are in the stage after recovery, and their poor physical condition may have a negative impact on work, resulting in cumulative fatigue (34). The findings of this study show a positive relationship between working hours and cumulative fatigue among health care professionals, which is consistent with previous research findings. The study revealed that the longer the working hours, the more severe the cumulative fatigue symptoms among primary health care professionals. As a result, health care system managers can improve by limiting working hours and reducing fatigue to reduce professionals’ cumulative fatigue symptoms. First and foremost, health care system managers should limit employees’ continuous working hours; for example, they should not work for 10 consecutive hours, plan employees’ shift schedules reasonably, allow rest at least 10 h between shifts, and reduce the number of unnecessary night shifts (29, 35). Studies have also shown that naps reduce workers’ fatigue, so health care managers can provide facilities for workers to take short breaks in the workplace (36). Furthermore, health care system managers can strengthen professionals’ health management, encourage them to do more physical exercise to improve their physical fitness, which can also relieve fatigue (37).

### 4.2. The mediating role of occupational stress

According to the findings of the study, 27.5% of primary health care professionals experience occupational stress, which means nearly one-third of all employees experience occupational stress. This is a phenomenon that health care administrators must be aware of. According to the findings of the analysis, there are statistically significant differences in the occupational stress levels of primary health care workers with different monthly incomes and occupations (*p* < 0.05). Occupational stress is more visible among primary health care workers who earn less than US$700 per month and are doctors. A previous researcher used the same COSS scale to measure occupational stress, and the detection rate was 27%, which is similar to this result (38). At the same time, another researcher used the Depression, Anxiety, and Stress Scale (DASS-21) Questionnaire to assess healthcare and administrative staff, and the probability of occupational stress is similar to ours, with a detection rate of 28.6% (39). The present study’s findings indicate that certain factors, such as low monthly income, occupational role as a doctor, and requirement of shift and night shift work, contribute significantly to the experience of occupational stress among primary health care workers. These findings align with previous research in the field. Furthermore, sustained occupational stress in this population can lead to mental exhaustion and physical symptoms, ultimately resulting in the development of cumulative fatigue (40). In this study, occupational stress serves as a mediator variable, mediating the relationship between working hours and cumulative fatigue of employees in the primary health care system. The analysis of the mediating effect results in Table 3 shows that occupational stress of primary health care employees plays a partial mediating role, with a 28.3% mediating effect percentage. As a result, by reducing occupational stress in the primary health care system, it is possible to reduce the cumulative fatigue symptoms of professionals caused by long hours of work.

The work of the primary health care system demands extensive professional skills and a high level of patient responsibility. Due to the medical industry’s extremely low tolerance for errors, employees must dedicate themselves to their work for extended periods. Even during non-working hours, professionals must engage in learning activities to enhance their professional ability, placing them under considerable occupational pressure. As a result, excessive investment of time and energy in their work can lead to fatigue. In comparison to medical and health personnel in developed countries, Chinese primary health care professionals have also dedicated a significant amount of time and energy. While the Chinese health industry’s salary and welfare levels still need to be improved (41). Some studies have confirmed that the disparity between investment and return is a significant factor influencing occupational stress (42). As a result, health care managers can implement a scientific performance appraisal and salary distribution system to try to achieve a balance between effort and reward, reduce occupational stress among primary health care workers, and relieve cumulative fatigue. Previous research has found that mindfulness meditation therapy is a potentially effective intervention for reducing occupational stress across various occupations. This form of therapy can enhance an individual’s attention and self-regulation abilities, ultimately improving cognitive, emotional, and behavioral functioning, and reducing psychological stress (43). When employees’ work tasks in the health system cannot be relieved, system managers can hire trained meditation teachers to provide collective training and guidance to primary health care professionals, thereby relieving occupational stress and reducing cumulative fatigue caused by long hours of work. Moreover, it is important for health care system managers to be mindful of their employees’ psychological well-being. They may engage professional psychotherapy teams to offer consultation services, and provide group mental health lectures to alleviate occupational stress and mitigate cumulative fatigue resulting from long hours of work. Managers of the health care systems can also offer regular health check-ups to detect early signs of physical illnesses among employees, provide logistical support to those who require it, and adjust workload and working hours to alleviate employees’ cumulative fatigue symptoms. The primary health care professionals should also exercise consciously to improve their physical fitness, and maintain a balance between work and rest in order to alleviate the symptoms of cumulative fatigue.

## 5. Conclusion and prospects

The present study aimed to examine the association between working hours, occupational stress, and cumulative fatigue symptoms in primary health care professionals. To summarize, working hours of the primary health care professionals would affect cumulative fatigue, and occupational stress could affect the influence of working hours on cumulative fatigue as a mediator variable. Long working hours result in occupational stress, which leaded to cumulative fatigue symptoms. Occupational stress was found to partially mediate the relationship between working hours and cumulative fatigue, with a mediating effect of 0.078 (95% CI: 0.043–0.115, *p* < 0.01), and the percentage of occupational stress mediating effect was 28.3%.To mitigate this, measures such as mindfulness meditation, group psychological counseling, health check-ups, improving salary systems, and rationally arranging working hours and shifts could be taken to reduce occupational stress and alleviate the symptoms of cumulative fatigue. Additionally, a mediating effect model was used to investigate the role of occupational stress as a mediator between working hours and cumulative fatigue symptoms. This study also offered practical recommendations and guidance to health care administrators, as well as a theoretical and practical foundation for workers’ occupational stress and cumulative fatigue.

For future studies, more longitudinal research is required to establish causal relationships. Furthermore, because this stud y only conducted surveys in Guangdong Province, China, it is necessary to be cautious when extrapolating the results to primary health care workers throughout the country. Future studies could expand the scope of the research by conducting surveys nationwide.

## Data availability statement

The raw data supporting the conclusions of this article will be made available by the authors, without undue reservation.

## Ethics statement

The studies involving human participants were reviewed and approved by the Medical Ethical Review Committee of the National Institute for Occupational Health and Poison Control of the Chinese Center for Disease Control and Prevention (protocol code NIOHP202108, 6/14/2022).

## Author contributions

YL: software, formal analysis, and original draft preparation. ZL: validation, formal analysis, and review and editing. QC and JW: investigation and resources. YF: data curation and investigation. YY and YC: resources and review and editing. TZ, LW, and YX: validation and review and editing. DZ: investigation, resources, and project administration. XY: validation, review and editing, and funding acquisition. All authors contributed to the article and approved the submitted version.

## Funding

The study was funded by the Special Investigation Project on Occupational Disease Hazards of Key Populations of the Occupational Health Institute of China CDC (131031109000160004), the Science and Technology Planning Project of Guangdong Province (2020B1212030008), Industry-Academia-Research Projects of Zhuhai City (ZH22017002200002PWC) and the Science and Technology Development Fund, Macau SAR (0024/2022/A).

## Conflict of interest

YY and YC were employed by Resproly Pharmaceutical Technology Co. Ltd.

The remaining authors declare that the research was conducted in the absence of any commercial or financial relationships that could be construed as a potential conflict of interest.

## Publisher’s note

All claims expressed in this article are solely those of the authors and do not necessarily represent those of their affiliated organizations, or those of the publisher, the editors and the reviewers. Any product that may be evaluated in this article, or claim that may be made by its manufacturer, is not guaranteed or endorsed by the publisher.
